# Learning from the past: inequalities and discrimination in psychiatry's chequered history

**DOI:** 10.1192/bjb.2021.68

**Published:** 2022-08

**Authors:** Claire Hilton, Robert Freudenthal

**Affiliations:** 1 Royal College of Psychiatrists, London, UK; 2 Enfield and Haringey Mental Health NHS Trust, London, UK

**Keywords:** Stigma and discrimination, history of psychiatry, Daniel Hack Tuke, Henry Maudsley, remembering the past

## Abstract

The Royal College of Psychiatrists’ antiquarian book collection originated from the library of psychiatrist Daniel Hack Tuke (1827–1895). A proposal to name the collection after him led us to investigate aspects of his life and work, particularly related to his attitudes concerning race, gender and homosexuality. We juxtaposed his ideas with those of some of his contemporaries. We cannot separate psychiatrists, past or present, from the societal and scientific context that shapes their professional understanding and standards. However, changes in language, knowledge, values and other sociocultural factors over time can affect how we perceive our forebears and how future generations of psychiatrists may perceive us.

The Royal College of Psychiatrists (RCPsych) published its Equality Action Plan in January 2021.^1^ It seeks to overcome inequalities that may have a negative impact on the well-being of patients and those who work with them. These inequalities, or ‘protected characteristics’, as designated by the Equality Act 2010, include race, religion, age, disability and the multiple facets of sexuality and gender. The Plan also celebrates many recent College equality achievements. Today, however, when the place of statues, memorials and legacies, particularly those associated with British colonialism, is being scrutinised,^2^ psychiatry needs to contextualise and understand aspects of its more distant past, as part of leading the way to create a more equal future.

For the RCPsych, 2021 also marks 50 years as a medical Royal College and 180 years since the founding of its first predecessor body, the Association of Medical Officers of Asylums and Hospitals for the Insane (AMOAHI). Stemming from discussions about how to celebrate this, the question arose as to whether the College's antiquarian book collection should be given a snappier name, to help raise its profile and encourage more people to use it. The collection began with 700 volumes donated by Esther Maria Tuke, the widow of psychiatrist Daniel Hack Tuke (1827–1895). Initially, it was the library of the Medico-Psychological Association (as the AMOAHI was renamed in 1865),^3^ so might we now call it the ‘Tuke Library’? This question led us to explore aspects of Daniel Hack Tuke's life and work, and those of some of his contemporaries. We drew on published work by 19th- and early 20th-century psychiatrists, most of which is available from the Internet Archive (www.archive.org) and in the *Journal of Mental Science (JMS,* predecessor of *British Journal of Psychiatry*). In this short essay we explore aspects of our Victorian psychiatrist forebears’ perspectives and understanding on matters of race, gender and homosexuality, in the sociocultural, knowledge and values framework of their own time. We also consider the difficulties of assessing figures from the past by reference to today's social values.

## Tuke and his contemporaries: asylums, homosexuality, women and degeneration

Daniel Hack Tuke was descended from William Tuke, who founded the Retreat at York in the 1790s. Based on the fundamental Quaker belief in the equality of all human beings, the Retreat aimed to provide more humane treatment for people with mental disorders than that available in the local public asylum. It was funded by donations from Quaker groups around the country, rather than from personal family wealth.

Hack Tuke, like his ancestors, pioneered humane social treatment. He worked for several years at the Retreat, and in 1879 was a founder member of the After-Care Association (today, Together for Mental Wellbeing). This organisation initially aimed to support women to re-establish their lives in the community following an asylum admission.^4^ Tuke co-edited the *JMS* for around 15 years, and undertook the mammoth task of editing the two-volume *Dictionary of Psychological Medicine* (1892).^5^ The *Dictionary* included articles by leading psychiatrists, psychologists and neurologists. It aimed to be a state-of-the-art scientific and clinical compendium. Relatively little of it directly addressed what today would be considered human rights or equality issues.

When trying to understand attitudes and behaviours of our forebears, we require a degree of scepticism when reading their obituaries, especially when written by colleagues, as these tend to follow the tradition of not speaking ill of the dead.^6^ It is unusual, however, to find one attributed to a patient, in this case Henry Francis Harding, which extols the virtues of a psychiatrist.^7,8^ Harding mentioned some of Tuke's personal attributes, which align with other evidence, to suggest that he maintained a good relationship with his son, the homosexual artist Henry Scott Tuke.^9^ In late Victorian England, this could not be taken for granted. In the *Dictionary*, Conolly Norman, medical superintendent of the Richmond District Lunatic Asylum, Dublin, contributed the entry on ‘sexual perversion’,^10^ one of the terms then used concerning homosexuality. He wrote empathetically, recognising the development of homosexual identity during childhood rather than it being a disorder that required treatment. Like Tuke, Norman also tried to provide the most humane treatment for his asylum patients. Also, unlike a significant number of their contemporaries, both of them were enthusiastic about supporting the election of women doctors to membership of the Medico-Psychological Association.^11^

In the second half of the 19th century, biological, hierarchical theories of mental, physical and cultural differences, which often put upper-class White men at the top, gained acceptance. Within the psychiatric literature, Bénédict Morel's degeneration hypothesis (1857)^12^ was prominent. It proposed a downward trajectory of health and well-being of individuals, families and society, if they bore a supposedly inherited taint. The degeneration hypothesis gained ground among novelists, the public, politicians, physicians and scientists, but it was probably less influential than might be expected among British psychiatrists. Although criticised for their lack of academic approach to psychiatry, many held a cautious scepticism towards hypotheses presented to them, a virtue in its own right and one that could protect the interests of their patients. Many were aware that their clinical observations were inconsistent with degeneration. They treated and discharged patients who were labelled as having ‘hereditary insanity’, and noted that their patients’ families did not show patterns of decline compatible with it.^13^ From the evidence available to us, Tuke and Norman were among those who tried to overcome ideas of biological hierarchies, hereditary predispositions, and inevitable decline and hopelessness associated with insanity. They tried to improve in-patient care and aftercare.^14^ They reached out beyond stereotypes and theories, and held liberal attitudes towards to people whose lives were affected negatively by social or professional exclusion.

## Language, race and weighing up the evidence

All historical sources require careful and contextualised analysis. Many Victorian psychiatrists left few private papers that might directly shed light on their lives as individuals. Their publications require cautious reading if one attempts to use them to infer personal attitudes or motivation for writing. For example, we can consider Havelock Ellis's (1897) writing on homosexuality (‘sexual inversion’). Unlike many of his contemporaries,^15^ he took a non-moralising, non-legalistic approach, explaining that ‘several persons for whom I felt respect and admiration were the congenital subjects of this […] instinct which to those persons who possess it frequently appears natural and normal’. However, to argue his case, some of the comparisons he made, such as with ‘the congenital idiot’ and ‘the man [*sic*] of genius’,^16^ and his language of normal and abnormal to stress human variation, make for uncomfortable reading today. Rather than looking at the individual words he used to assist his expected readership to understand his argument, we need to consider the take-home message.

The meaning of many words used by previous generations has shifted. Language with an emotional overlay and associated with stigma changes particularly rapidly. Earlier professional and official terminology, such as cripple, spastic, lunatic and imbecile, have become terms of abuse. ‘Race’ has also changed. In its broadest meaning there is the ‘human race’. In Tuke's time, race often indicated difference or foreign-ness rather than specifically skin colour, such as the ‘Jewish race’, then used in popular literature^17^ and in medical parlance.^18^ Tuke tried to weigh up the evidence presented to him. He wrote of uncivilised, savage or barbarian nations, but associated their lifestyle with good mental health. However, he also acknowledged that his evidence was based on ‘the testimony of travellers’, so interpreting it required ‘extreme caution’. Nevertheless, he wrote that it ‘must not be disregarded, but be accepted as the nearest approach we can make’.^19^ He also criticised lifestyles and leadership in his own country for conditions that, in his view, increased insanity, such as the ‘curse of civilised pauperism’ and the ‘widespread tippling which disgraces England’.^20^ Tuke was not one-sided in his interpretation of culture or race as it related to mental well-being. In his view, life in England was more likely to cause mental disorders than life in distant countries. Tuke exemplified a psychiatrist who sought a scientific understanding of mental disorders while recognising limitations and uncertainties of the evidence available to him.

Tuke's ideas contrast with those of his more famous colleague, Henry Maudsley.^21^ Maudsley probably destroyed his private papers,^22^ so we know little about him as a person, although his own published writings, and what his contemporaries wrote about him, provide clues. In 1867, in the context of discussing the evolution of brain and mind, Maudsley wrote:

‘The brain of the Negro is superior to that of the Bushman, but still it does not reach the level of the white man's brain; the weight of the male Negro's brain is less than that of the average European female; and the greater symmetry of its convolutions, and the narrowness of the hemispheres in front, are points in which it resembles the brain of the ourang-outang, as even Tiedemann, the Negro's advocate, has admitted.’^23^

Frederick Tiedemann (Professor of Anatomy and Physiology, Heidelberg) compared orangutan and human brains in the 1830s. He found only minute differences between brains of Black and White people, with any resemblance to those of other primates of dubious significance. He concluded that ‘neither anatomy nor physiology can justify our placing [Black people] beneath the Europeans’ morally or intellectually, and that any differences were the result of oppression and the cruelty of slavery.^24^ However, almost 40 years after Tiedemann published his research, Maudsley misrepresented it, advocating instead a theory affirming racial difference.^25^ As is common for research that contradicts established theories or cultural, political or economic drivers, Tiedemann's research had little impact. In this case, it conflicted with elitist ideals of Empire, and Maudsley accepted it uncritically.

In 1896, an anonymous review of Maudsley's *The Pathology of Mind* appeared in the *JMS*. It criticised both book and author:

‘[his] philosophy is frequently unsound, his psychology prohibitive of truth, and his sociology repulsive and unsuited to average humanity … His rhetorical and precise statements on questions of a highly controversial nature seem to us unscientific and misleading ….They are out of sympathy with the general tenor of recent research and philosophic thought.’^21^

Considering that Maudsley was a former editor of the *JMS*, it is astounding that this, and a string of other criticisms, appeared in the *Journal*.^21^ His ideas were intolerable to his peers, who regarded them as behind the times and unworthy of debate. According to Trevor Turner, Maudsley's heritage also included ‘the dangers of drawing philosophy from clinical experience, the sterility of pure materialism in the face of human needs, the easy lapse into unscientific thinking’.^26^ Remembered, or perhaps glorified, by his enormous donation to the London County Council to establish the hospital that bears his name, there were other faces to a man we know little about.^27^

## Discussion

In the Biblical story of Noah's Ark, Noah was saved from the flood as he was ‘a righteous man, upright in his generation’ (Genesis 6:9). He was not perfect, or saint like, but the best in his time. Similarly, we need to understand our psychiatric forebears in the context of the era in which they lived. On the basis of our historical analysis, Tuke was not perfect, and some of the words he used may be contentious, but it would be hard to describe him as racist, homophobic or misogynistic. He tackled difficult and disputed subjects that lay on the fringes of societal and professional understanding, and acted to support people to fulfil their potential. This contrasted with Maudsley and his ideas, scorned by colleagues. We read critically the work of others, such as Charles Dickens, who used language similar to Tuke's, and we delve into his writings to understand the context, whys and wherefores of his motivations, experiences and meaning. We need to do the same for our forebears in psychiatry.

Disparity between the language used and the action it engenders is important. Today, the language of ‘third world’ and ‘first world’ or ‘developing’ and ‘developed’ countries (which expressed so-called ‘progress’ towards becoming materially wealthier industrialised Westernised societies) has fallen from favour. Today's recommended language of low-, middle- and high-income countries rings more kindly to our ears, but this does not necessarily change actions or prevent perpetuation of inequalities. The distribution of COVID-19 vaccine is an example in point.^28^ Changing the words we use does not equate with changing practice. We are likely to consider someone who uses the terms ‘third world’ or ‘developing country’ as ill-informed rather than racist. Taking words out of context can lead to unwarranted assumptions. Since uncomfortable language changes rapidly, Tuke's words are inevitably outdated. Tuke was ill-informed and aware that he lacked knowledge, but sought better and tried to make sense of the evidence presented to him.

In the context of current debates on discrimination, the RCPsych will not be naming the antiquarian book collection after Tuke. This is understandable in the context of implementing the College's Equality Action Plan^1^ and wanting to avoid anything that might possibly be (mis)interpreted as having racist connotations. However, imposing today's standards about equality onto a different culture and generation is ahistorical. It also sets a precedent for future generations to regard our theories and practices as unacceptable, despite us trying to do our best for our patients amid imperfect social, cultural, political, economic and scientific circumstances. How, for example, might future generations interpret the high rates of detention of Black men under the Mental Health Act 1983?^29^ As Mind's *Legal Newsletter* asked: ‘Could it be that stereotypes of black people, men especially, as being dangerous are operating at a sub-conscious level on decision-makers at the point of sectioning?’^30^ Will we be described as racist, or as psychiatrists trying our best in a less than perfect world? We cannot separate psychiatrists, past or present, from the societal and scientific context that shapes their professional understanding and standards. In addition, ahistorical analyses risk generating complacency. If future generations of psychiatrists see the past as inevitably flawed and themselves as inevitably better than their predecessors, they will fail to use history to question their own practices and to learn from what happened before them.

A short article can only begin to unravel the history of attitudes and values of Victorian psychiatrists, and the subject would benefit from more investigation. Psychiatry has skeletons in the cupboard, but we must not write our predecessors out of history on the basis of flimsy evidence or interpretation of words taken out of their historical context. The lives and teachings of our psychiatric predecessors require critical, contextualised historical analysis rather than rejection. They were not perfect, neither was Noah and nor are we.

## Data Availability

Data availability is not applicable to this article as no new data were created or analysed in this study.

## About the authors

**Claire Hilton**, MD, PhD, FRCPsych, is Historian in Residence at the Royal College of Psychiatrists, London, UK. **Robert Freudenthal**, MRCP, MRCPsych, MSc, is a Specialty Trainee Year 6 (ST6) psychiatrist at St Ann's Hospital, Barnet, Enfield and Haringey Mental Health NHS Trust, London, UK.
